# Provider compliance to artemisinin-based combination therapy at primary health care facilities in the middle belt of Ghana

**DOI:** 10.1186/s12936-015-0902-1

**Published:** 2015-09-22

**Authors:** Anthony Kwarteng, Kwaku Poku Asante, Livesy Abokyi, Stephaney Gyaase, Lawrence G. Febir, Emmanuel Mahama, Dennis G. Konadu, Theresa Tawiah, Dennis Adu-Gyasi, David Dosoo, Seeba Amenga-Etego, Bernhards Ogutu, Seth Owusu-Agyei

**Affiliations:** Kintampo Health Research Centre, Ghana Health Service, P. O. Box 200, Kintampo, Ghana; INDEPTH Network, Accra, Ghana

**Keywords:** Malaria, Health worker, Provider compliance, National guidelines, Artemisinin-based combination therapy, Middle Ghana

## Abstract

**Background:**

In 2004, Ghana implemented the artemisinin-based combination therapy (ACT) policy. Health worker (HW) adherence to the national malaria guidelines on case-management with ACT for children below 5 years of age and older patients presenting at health facilities (HF) for primary illness consultations was evaluated 5 years post-ACT policy change.

**Methods:**

Cross-sectional surveys were conducted from 2010 to 2011 at HFs that provide curative care as part of outpatient activities in two districts located in the middle belt of Ghana to coincide with the periods of low and high malaria transmission seasons. A review of patient medical records, HW interviews, HF inventories and finger-pricked blood obtained for independent malaria microscopy were used to assess HW practices on malaria case-management.

**Results:**

Data from 130 HW interviews, 769 patient medical records at 20 HFs over 75 survey days were individually linked and evaluated. The majority of consultations were performed at health centres/clinics (68.3 %) by medical assistants (28.6 %) and nurse aids (23.5 %). About 68.4 % of HWs had received ACT-specific training and 51.9 %, supervisory visits in the preceding 6 months. Despite the availability of malaria diagnostic test at most HFs (94 %), only 39.8 % (241) out of 605 (78.7 %) patients who reported fever were investigated for malaria. Treatment with ACT in line with the guidelines was 66.7 %; higher in <5 children compared to patients ≥5 years old. Judged against reference microscopy, only 44.8 % (107/239) of ACT prescriptions that conformed to the guidelines were “truly malaria”. Multivariate logistic regression analysis showed that HW were significantly more likely to comply with the guidelines if treatment were by low cadre of health staff, were for children below 5 years of age, and malaria test was performed.

**Conclusion:**

Although the majority of patients presenting with malaria received treatment according to the national malaria guidelines, there were widespread inappropriate treatment with ACT. Compliance with the guidelines on ACT use was low, 5 years post-ACT policy change. The Ghana NMCP needs to strengthen HW capacity on malaria case-management through regular training supported by effective laboratory quality control measures.

## Background

Significant progress has been made in the last decade towards achieving global malaria targets although the disease burden is still relatively high in sub-Saharan African countries, especially among children <5 years of age [1, 2]. According to a recent report by the World Health Organization (WHO), malaria accounted for approximately 1800 admissions and 10 deaths for every 100,000 population in Ghana [2].

Prompt and effective malaria case management has been the main control strategy [3, 4]. Ghana implemented the artemisinin-based combination therapy (ACT) policy in 2004 with artesunate–amodiaquine as the first-line drug to replace chloroquine which is no more effective due to widespread development of *Plasmodium* resistance across Africa [5]. The anti-malarial drug policy of ACT was revised in 2009 to include artemether–lumefantrine and dihydroartemisinin–piperaquine as alternative first-line treatment drugs due to poor tolerability of artesunate–amodiaquine by sections of the Ghanaian community [6]. The successful implementation of an ACT policy to a large extent depends on the availability of the ACT drugs and the ability of health professionals to adhere to national malaria treatment guidelines [7, 8]. The introduction of Affordable Medicine Facility for Malaria (AMFm) to subsidize ACT, has expanded access to ACT and gave hope to endemic countries that piloted the scheme [9, 10].

Ghana revised her national guidelines for malaria case management to address changes in the anti-malarial drug policy [11]. The policy recommends parasitological diagnosis (using malaria rapid diagnostic test or microscopic examination of malaria blood smear) of all suspected cases prior to treatment with ACT. Specifically, the policy allowed presumptive diagnoses and treatment for malaria for children below 5 years due to operational considerations and the fact that Ghana is a high malaria risk area [12]; but with a long-term goal of deploying a test, treat and track policy of all suspected cases. Treatment with ACT is recommended for positive test results for all children and adults except for pregnant women in the first trimester who are required to use quinine.

Restricting treatment to parasitological diagnosis of malaria among older children and adults is expected to reduce over-diagnosis of malaria and inappropriate use of relatively expensive ACT in the treatment of fevers [13]. Proper clinical interpretation based on test results remains the main challenge in a change process after many years of believing that presence of fever denotes malaria [14].

In this study, the quality of uncomplicated malaria case management for children <5 years and older patients presenting for primary illness consultation was assessed at health facilities (HFs) in two districts of the middle belt of Ghana 5 years post**-**ACT policy implementation by evaluating health worker (HW) compliance to the national malaria guidelines on ACT prescriptions.

## Methods

### Study area

The study was conducted in Kintampo North and South Districts, which lie within the forest savannah transitional ecological zone in the middle belt of Ghana. The Kintampo districts cover an area of 7162 km^2^ and has a resident population of 134,970 as at 2009 [15]. Subsistent farming is the main economic activity. Mean monthly temperature ranges between 18 and 38 °C with average rainfall of 1250 mm per annum. In children below 5 years of age, the incidence of malaria annually is seven episodes per child per year [16]. Malaria transmission is high (entomological inoculation rate: 269 infective bites per person per year) throughout the year but peaks between May and October and dips between December and April [17]. Like in other parts of Ghana, majority of malaria cases are diagnosed and treated at the community and/or the primary health facility levels [11]. Licensed chemical shops are the first point of care for many illnesses including malaria [18]. There are two district hospitals that serve as patient referral points, 12 health centres/clinics and 30 community-based health planning services (CHPS) compounds. At the hospitals and private clinics, parasitiological diagnosis of malaria are often by malaria microscopy whilst malaria RDT kits are used at the peripheral HFs.

### Study design

The design was a cross-sectional HF survey involving review of patient medical records, HW interviews, HF inventory and independent blood slide reading by a reference laboratory to assess provider compliance to malaria case-management based on the Ghana national malaria treatment guidelines.

### Sample size calculation for health facility survey

In a controlled trial carried out in southern Ghana compliance to malaria management with ACT based on malaria RDT and blood slide was 60.1 and 57.1 %, respectively [19]. Seven hundred and twenty patients presenting for primary illness consultation were required to be sampled from at least 31 clusters with an intra**-**class correlation 0.2 to be able to detect an assumed lesser compliance of ≤53 % in this non-controlled study with 95 % confidence and with 90 % statistical power. A cluster was defined as all patient consultations performed at a HF on 1 day during regular working hours from 8 am to 5 pm. A primary illness consultation was defined as the first time a patient visited the HF for the treatment of the presenting illness episode.

### Health facility surveys

Three cross-sectional cluster surveys each lasting 5–8 weeks were conducted in January 2010, June 2010 and in January 2011 at HFs that provide curative care as part of outpatient activities. These surveys were planned to coincide with the periods of low (January 2010 and January 2011) and high malaria transmission seasons (June 2010). Data collection was undertaken by a team comprising of a clinician, a laboratory technician and field data collectors under the supervision of the study investigators.

#### Selection

A list of 19 HFs in the study area that provided curative care as part of an out-patient setting during regular working hours from 8 am to 5 pm was used as the sampling frame. The HFs in the sampling frame increased to 20 with the establishment of an additional HF before the commencement of the second survey. Using a written programme in STATA, the HFs were randomly ordered and numbered consecutively. The HFs were assigned to pre-determined survey dates and visited in the same order and date as pre-determined. No other facility was visited on a survey day when the scheduled facility was found closed. HFs closed on the date of the survey were visited during a “catch-up period” on the same day of the week as originally determined. Dates of visits were not announced in advance to HF staff. On arrival at each HF, all HWs performing patient consultations were assigned identification numbers. Using a structured questionnaire, HWs were interviewed on their most recent ACT specific training, supervisory visits received and knowledge of the national malaria treatment guidelines. An inventory of diagnostic test kits, ACT and other medications available at the HF was made before patient consultations began.

#### Enrolment

On a survey day, all patients at the HF who met the inclusion criteria of seeking primary consultation for their illness were asked to provide informed consent for participating in the study. After receiving treatment, patients who had given an informed written consent had their weight and axillary temperature re-assessed using standardized equipment by the survey team. Patient demographics, vital signs, medical records and insecticide treated net (ITN) use were recorded on a standardized questionnaire. About 0.5 µl of whole blood was collected into EDTA (ethylenediaminetetraacetic acid) tubes and stored in a cool box and transferred to the reference laboratory for malaria microscopy. A review of patient medical records was performed by the team leader to extract medical information of the care patient received on the survey day. Information extracted from the patient folders included HWs’ record of patient medical history, presenting symptoms, diagnosis and treatment.

### Blood sample processing at the reference laboratory

Thick and thin malaria films were prepared from blood collected and stained with Giemsa using the method recommended by WHO for blood smear preparation. The malaria blood smears were read independently by two experienced microscopists. In cases where the results were discrepant, a third microscopist read the slide for concordence with one of the two earlier readers. Assuming a count of 8000 leukocytes/dl of blood, parasite densities were calculated by counting the number of asexual parasites per 200 white blood cells [20]. A blood smear was considered negative if no parasite was found after counting 200 high power fields.

### Data management and statistical analysis

All data forms were checked manually for completeness and consistencies before data processing. Epi info version 7 software package was used for double entry, validation and verification of all data. Analysis was carried out using STATA, version 11 (StataCorp, College Station, TX, USA). The quality of outpatient malaria case management for each patient was evaluated by individually linking patient data (extracted medical information and reference malaria blood slide results) to the information of the HW and HF where patient received care on the survey day. The availability or absence of diagnostic test kits, medicines and ACT-specific training on the quality of care was further assessed.

The primary outcome for assessment was provider compliance based on the case management guidelines of the Ghana NMCP otherwise referred to as appropriate ACT treatment [11]. For all age groups, provider compliance was defined as the proportion of patients presenting at a HF for primary illness consultation with or without fever but in the absence of any sign of severe malaria who were prescribed ACT on a positive test for malaria parasites or had an ACT withheld when a test for malaria parasite was negative. Among patients aged <5 years however, a diagnosis of malaria based on confirmatory testing was not required. A patient was considered to have fever if he/she had a record of fever within the previous 48 h and/or axillary temperature ≥37.5 °C.

A comparative analysis of HW routine use and interpretation of malaria diagnostic test for <5 children and older patients with respect to a presentation of fever and ACT prescriptions was performed. The sensitivity, specificity as well as positive and negative predictive values (NPV) of routine diagnostic tests relative to the reference microscopy were determined.

Univariate analysis was used to identify predictors of provider compliance to the national malaria treatment guidelines. Seasonality of malaria transmission and other explanatory variables such as age of patient, mode of diagnosis, type and level of HF, HW cadre and training on IMCI that showed a tendency of association with provider compliance were included in the multivariate logistic regression. Data was summarized as frequencies and proportions with corresponding 95 % confidence interval adjusted for clustering by HF.

### Ethical consideration

Ethical approval was provided by the Ghana Health Service Ethics Review Committee (GHS-ERC) and Kintampo Health Research Centre Institutional Ethics Committee (KHRC-IEC). Permission to conduct the HF surveys were obtained from the local health administrations. Informed written consent was obtained from patients and caretakers of children who were willing to participate in the study. Children aged 12–17 years of age were requested to provide their assent in addition to consent by parent or accompanying adult without which a patient was excluded from the study. Unaccompanied minors were excluded as per regulations of the KHRC-IEC. Informed written consent were also obtained from HWs who performed patient consultation on the day of survey. To maintain confidentiality, a unique study number/code was assigned to each participant as an identifier at enrolment for labelling all blood samples and study forms. All study data were stored securely with restricted access.

## Results

### Description of the sample population

**Fig. 1:**
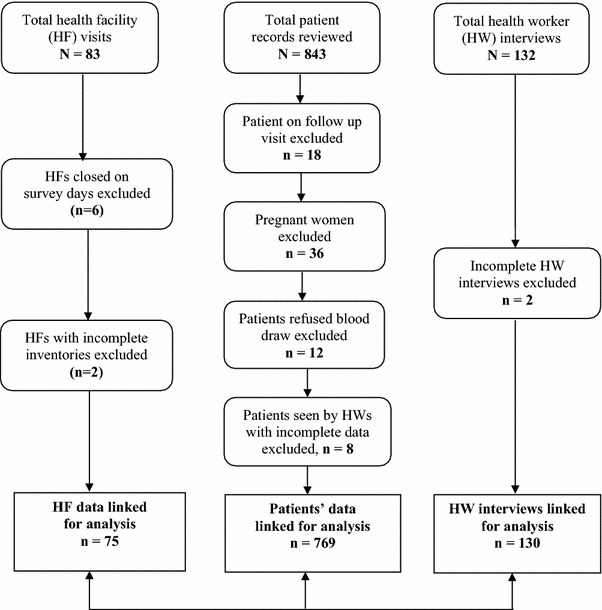
Description of sample population and data analyzed

Overall, 843 patient folder reviews and 132 HW interviews were performed at 20 HFs through 83 HF visits during three cross-sectional surveys (Fig. 1). In the first survey, 19 HFs were each visited once during which 143 patient folder reviews and 42 HW interviews were conducted. The second survey involved visits to 20 HFs during which 243 patient folder reviews and 44 HW interviews were performed. During both surveys, one HF that was closed on the survey day was still not opened during the “catch-up period”. To increase patient enrolment during the third survey, each of the 20 HFs was visited twice during which 457 folder reviews and 46 HW interviews were performed. Two HFs that were closed on the first visit were available for study activities during the “catch-up period”.

Data from eight HF visits were excluded because they were closed (6) or had incomplete inventories (2) on the day of the visit. Seventy-four patient records were excluded from the analysis. Those excluded comprised of 18 patients who were on follow-up visits for previous illness and 36 pregnant women on the basis that the national guidelines does not recommend ACT treatment for pregnant women in the first trimester and the team did not plan for gestational assessment as part of this study. Others were 12 patients who did not consent to blood draw and 8 patients seen by two HWs whose interviews were incomplete. Analysis was, therefore, carried out on 769 patients’ data individually linked with each of the 130 HWs and 75 facility information of where patient received care on each survey day.

### Background patient consultation by facility and health worker

The proportion of children <5 years of age presenting for a primary illness consultation was higher during the high malaria transmission season (HMTS) (33.8 %) than the low malaria transmission season (LMTS) (26.1 %) (Table 1). Insecticide-treated nets (ITNs) use the night before the survey was 34 % in the low LMTS and 51.4 % in the HMTS. The prevalence of fever was 70.7 % in the LMTS and 100 % in the HMTS. The seasonal difference in fever prevalence was statistically significant (p < 0.001) (Table 1).

**Table 1:** Characteristics of patient presenting for initial consultations during the low and high malaria transmission seasons. N = 769

Characteristics	Low season N = 559 n (%)	High season N = 210 n (%)	Both seasons N = 769 n (%)
Median age in years (range)			
<5 year olds	146 (26.1)	71 (33.8)	217 (28.2)
≥5 year olds	413 (73.9)	139 (66.2)	552 (71.8)
Female	349 (62.4)	117 (55.7)	466 (60.6)
Used ITN the previous night	190 (34.0)	108 (51.4)	298 (38.8)
NHIS insured	509 (91.1)	186 (88.6)	695 (90.4)
Fever prevalence	395 (70.7)	210 (100.0)	605 (78.7)

*ITN* insecticide-treated net, *NHIS* National Health Insurance Scheme

A majority of patients (68.3 %; 525/769) were seen at the health centres/clinics (Table 2). Most patients were seen by medical assistants (28.6 %) and nurse aids or lower cadres (23.5 %) while medical doctors performed 18.7 % of patient consultations in both seasons (Table 2). HWs trained on the malaria treatment guidelines were 68.4 and 51.9 % had received supervisory visits in the 6 months preceding survey. Functioning thermometer and weighing scales, diagnostic tools (microscopy or RDT) and at least any one of the recommended ACT were frequently available at the HFs during patient consultations (Table 2). However, only 45.1 % of patients were seen in consulting rooms where the revised ACT or the national malaria treatment guidelines was available (Table 2).

**Table 2:** Background health facility and health worker characteristics of patient consultations during the low and high malaria transmission seasons. N = 769

Characteristics	Low season N = 559 n (%)	High season N = 210 n (%)	Both seasons N = 769 n (%)
Health facility characteristics			
Type of facility			
Hospital	117 (20.9)	37 (17.6)	154 (20.0)
Health centre/clinic	379 (67.8)	146 (69.5)	525 (68.3)
CHPS	63 (11.3)	27 (12.9)	90 (11.7)
Functional equipment			
Weight scale for all ages	13 (2.3)	66 (31.4)	79 (10.3)
At least one weight scale	559 (100.0)	210 (100.0)	769 (100.0)
Thermometer	555 (99.3)	210 (100.0)	765 (99.5)
Wall charts/reference material			
Wall flowchart	494 (88.4)	172 (81.9)	666 (86.6)
Revised malaria treatment guidelines	250 (44.7)	97 (46.2)	347 (45.1)
Diagnostic testing available			
Microscopy	344 (61.5)	113 (53.8)	457 (59.4)
Rapid diagnostic testing	348 (62.3)	170 (81.0)	518 (67.4)
Either microscopy or RDT	520 (93.0)	203 (96.7)	723 (94.0)
Anti-malarial stock			
Artesunate amodiaquine	486 (86.9)	210 (100.0)	696 (90.5)
Artemether lumefantrine	465 (83.2)	173 (82.4)	638 (83.0)
Dihydroartemisinin piperaquine	97 (17.4)	0	97 (12.6)
At least an ACT	559 (100.0)	210 (100.0)	769 (100.0)
Health worker characteristics			
Cadre consulting			
Medical doctor	102 (18.3)	42 (20.0)	144 (18.7)
Medical assistant	178 (31.8)	42 (20.0)	220 (28.6)
Midwife	47 (8.4)	15 (7.1)	62 (8.1)
Trained nurse	47 (8.4)	30 (14.3)	77 (10.0)
CHN	62 (11.1)	23 (11.0)	85 (11.1)
Nurse aid or lower	123 (22.0)	58 (27.6)	181 (23.5)
Training			
ACT-specific	383 (68.5)	151 (71.9)	534 (68.4)
IMCI	135 (24.5)	29 (13.8)	164 (21.3)
Supervision within previous 6 months	289 (51.7)	110 (52.4)	399 (51.9)

N = 769

*CHPS* community health planning services, *CHN* community health nurses, *IMCI* integrated management of childhood illness

### Evaluating health worker practices against the national malaria treatment guidelines

**Fig. 2:**
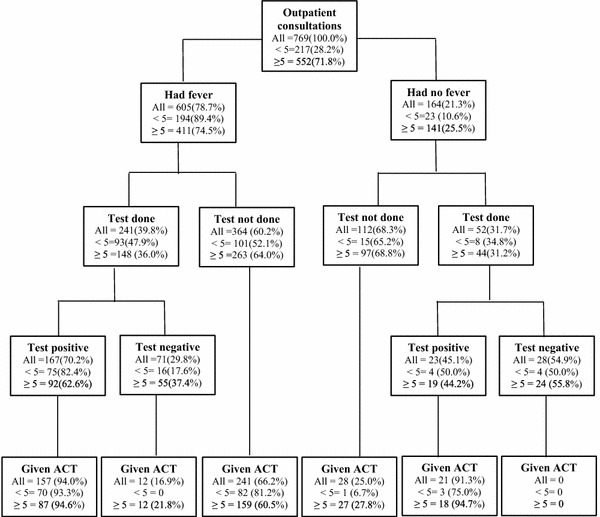
Health worker routine use and interpretation of malaria diagnostic test for <5 children and older patients with respect to a presentation of fever and ACT prescriptions. HW compliance to the national malaria treatment guideline for all patients was 66.7 %; 85.9 % in <5 children and 59.7 % in older patients

Figure 2 shows HW routine practice on the use and interpretation of diagnostic test results with respect to the presentations of fever and ACT prescription. The proportion of patients presenting for primary illness consultation without signs of severe malaria who reported a history of fever or an axillary temperature ≥37.5 °C was 78.7 % (605/769), higher among <5 children (89.4 %; 194/217) than older patients (74.5 %; 411/552).

#### Use and interpretation of malaria diagnostic test

Overall, only 39.8 % (241/605) of patients who presented with fever had a malaria diagnostic test (RDT or microscopy) performed; 47.9 % in children <5 and 36.0 % in patients ≥5 years. Of patients with fever who had a malaria test performed, 70.2 % (167/238) had malaria positive results more commonly in <5 children (82.4 %) than older patients (62.6 %). Among the 164 patients who had no report of fever, 52 (31.7 %) had a malaria test performed of which 45.1 % (23/52) were positive; marginally higher in children <5 years (50 versus 44.2 %).

#### Prescription of ACT

The prescription of ACT for patients who had fever and malaria parasite was high (94 %; 157/167), 93.3 % in children <5 years and 94.6 % in patients ≥5 years. Furthermore, presumptive treatment with ACT for febrile children <5 years was very high (81 %; 82/101). Contrary to treatment recommendation, 159 (60.5 %) of patients ≥5 years who presented with fever were prescribed ACT without a malaria test performed.

Among 164 patients presenting with no fever, 31.7 % (52/164) were tested. Of these, 45.1 % (23/52) were positive and 91.3 % (21/23) were treated. No treatment was given to those found negative.

#### Measuring provider compliance

In computing the primary outcome, 16 children <5 years who presented with fever but had a negative malaria test were excluded. This was done to prevent judging as incorrect HW practice of prescribing ACT for <5 children who had a negative malaria test as the national treatment guidelines states that *in <5 children, fever or a history of fever in the absence of other causes of fever should be considered malaria and treatment commenced immediately without waiting for laboratory results.* Three febrile children whose malaria test were not documented were also excluded.

Of the 750 patients presenting at HFs for primary illness consultation, 66.7 % were treated in accordance with the national malaria treatment guidelines; 239 received ACT whilst ACT were appropriately withheld in 261 patients. About 89.2 % (239/268) of <5 children presenting with at least fever and patients ≥5 years with uncomplicated malaria were correctly treated with ACT. ACT correctly withheld in both <5 children who had no fever and older patients who had no routine diagnosis of uncomplicated malaria was 54.2 % (261/482). Judged against results from the reference microscopy, however, only 44.8 % (107/239) of routinely prescribed ACT that conformed to the malaria guidelines were “truly malaria”. On the other hand, 8 % (21/261) of patients missed the opportunity to receive ACT during routine care due to either false negative results.

### The diagnostic accuracy of routine testing

The accuracy of the routine diagnostic tests namely malaria RDT and microscopy was determined with reference to expert microscopy (Table 3). The sensitivity of RDTs and microscopy were 67.4 and 34.2 % respectively. The specificities of routine diagnostics were high >95 % (RDT, 98.3 % and microscopy, 96.0 %). The positive predictive value (PPV) was high >97.5 % for both routine RDT and microscopy whilst the negative predictive value (NPV) was very low for routine microscopy (23.3 %) and relatively higher for RDT (66.7 %).

**Table 3:** The diagnostic accuracy of routine testing compared with expert microscopy

Prevalence	Rapid diagnostic test	Microscopy
	n/N (%; 95 % CI)	n/N (%; 95 % CI)
Sensitivity	60/89 (67.4; 56.7–77.0)	41/120 (34.2; 25.8–43.4)
Specificity	58/59 (98.3; 90.9–100.0)	24/25 (96.0; 79.6–99.9)
Positive predictive value	60/61 (98.4; 91.2 –100.0)	41/42 (97.6; 87.4–99.9)
Negative predictive value	58/87 (66.7; 55.7–76.4)	24/103 (23.3;15.5–32.7)

### Factors associated with health worker compliance to treatment guidelines

When univariate analysis was carried out, HW compliance in public HFs was found to be 66 % higher compared to private HFs. CHPS were 92 % more likely to conform to treatment guidelines compared to hospitals. Compared to medical doctors, less qualified HWs such as midwives, community health nurse (CHN) and nurse aids were 4.74 times (OR = 4.74, 95 % CI 2.10–10.72); 3.17 times (OR = 3.17, 95 % CI 1.65–6.10) and 1.69 times (OR = 1.69, 95 % CI 1.07–2.69) more likely to conform to treatment guidelines.

Results of the multivariate logistic regression shows that mode of diagnosis for patients, age group and the cadre of HW were significantly associated with HW compliance to the national malaria treatment guidelines (Table 4). A clinical decision based on presumptive diagnosis to prescribe or withhold ACT to a patient suspected with malaria on a presentation of fever in the absence of any signs of severe malaria was 69 % (OR = 0.31, 95 % CI 0.15–0.62) less likely to conform to the guidelines compared to one that was based on parasitological diagnosis. HW management of children <5 years were significantly over four times (OR = 4.49, 95 % CI 2.85–7.08) more likely to conform to the treatment guidelines compared to patients ≥5 years. The likelihood of less qualified HWs adhering better to the guidelines were demonstrated by community health nurse and midwives respectively 2.95 times (OR = 2.95, 95 % CI 1.37–6.38) and 4.87 times (OR = 4.87, 95 % CI 1.99–11.08) at higher odds of complying with the guidelines compared to medical doctors. The differences were statistically significant.

**Table 4:** Factors associated with health worker compliance to treatment guideline. N = 750

Factors	n (%) complied	Univariate logistic regression			Multivariate logistic regression		
		OR	95 % CI	p value	OR	95 % CI	p value
Malaria transmission season							
Low	65.81	1			1		
High	68.93	1.15	0.82–1.63	0.418	0.99	0.68–1.46	0.996
Mode of diagnosis							
Parasite based	67.61	1			1		
Presumptive	52.17	0.52	0.29–0.95	0.034	0.31	0.15–0.62	0.001
Age category of patient							
≥5 years	59.71	1			1		
<5 years	85.93	4.12	2.67–6.36	0.000	4.49	2.85–7.08	0.000
Active member of the National Health Insurance Scheme							
Yes	65.68	1					
No	76.06	1.65	0.94–2.93	0.080	N/A	N/A	N/A
Type of health facility							
Private	59.87	1			1		
Public (MOH)	71.18	1.66	1.23–2.25	0.001	1.25	0.80–1.97	0.328
Level of health facility							
Hospital	65.13	1			1		
Health centre/clinic	65.17	1.00	0.68–1.46	0.994	0.66	0.37–1.16	0.148
CHPS	78.16	1.92	1.04–3.52	0.036	0.99	0.45–2.19	0.976
Cadre of health worker							
Medical doctor	57.34	1			1		
Medical assistant	60.47	1.14	0.74–1.75	0.556	0.97	0.61–1.54	0.900
Trained nurse	64.94	1.38	0.78–2.44	0.274	1.62	0.81–3.23	0.169
Nurse aide or lower	69.49	1.69	1.07–2.69	0.025	1.61	0.88–2.94	0.124
Community health nurse	81.01	3.17	1.65–6.10	0.001	2.95	1.37–6.38	0.006
Midwife	86.44	4.74	2.10–10.72	0.000	4.87	1.99–11.08	0.001
Health worker received any ACT-specific training							
Yes	67.82	1					
No	64.04	0.84	0.61–1.17	0.313	N/A	N/A	N/A
Availability of malaria diagnostic tools							
Yes	67.50	1					
No	61.68	0.77	0.51–1.18	0.238	N/A	N/A	N/A
Availability of wall flowchart in the consulting room							
Yes	66.10	1					
No	70.30	1.21	0.77–1.92	0.406	N/A	N/A	N/A
Availability of revised malaria treatment guidelines in the consulting room							
Yes	69.85	1					
No	64.10	0.77	0.57–1.05	0.097	N/A	N/A	N/A
Health worker attended any training on Integrated Management of Childhood Illness							
Yes	73.91	1			1		
No	64.69	0.65	0.44–0.96	0.028	0.66	0.42–1.05	0.079
Health worker had any supervisory visits within 6 months preceding the survey							
Yes	66.58	1					
No	66.76	1.01	0.74–1.37	0.959	N/A	N/A	N/A

N = 750

*N/A* non applicable

## Discussion

In this study, 66.7 % of patients presenting at HFs for primary illness consultation received treatment that was in line with the 2009 national malaria case-management guidelines 5 years after the deployment of the ACT policy in Ghana. Appropriate treatment with ACT was significantly higher in <5 children (85.9 %) than in older patients (59.7 %). The prescription of ACT based on a correct diagnosis of malaria as indicated by the national guidelines was high (89.2 %). However, 45 % of patients received ACT based on a wrong diagnosis of malaria. As a result of the low sensitivity of routine test, 8 % of patients who were confirmed by reference microscopy to have malaria missed the opportunity for any anti-malarial treatment.

HWs non-compliance with the guidelines was a major cause of over-treatment with ACT. HW non-compliant behaviour observed in this study is similar to results from southern Ghana [19] and across many malaria endemic African countries namely Kenya [21], Angola [22], Zambia [23], Benin [24] and The Gambia [25] after anti-malarial policy change.

The lower compliance with ACT treatment based on presumptive diagnosis with its attendant over-treatment justifies the transition to new guidelines by the WHO requiring parasitological diagnosis of malaria universally regardless of age and malaria transmission levels (Table 4). Unfortunately, despite the universal availability of malaria diagnostic tools such as RDT and microscopy during patient consultations, they were often not used to guide treatment [26] neither were existing guidelines restricting ACT to only positive test in older patients complied with [27].

HW disregard to the existing guidelines raises questions about HW attitude and confidence in test results. For example, about 17 % of patients ≥5 years who presented with fever but had a negative test of malaria received ACT. Arguably, the unacceptably low sensitivity and NPV of routine diagnostics especially malaria microscopy compared to the reference microscopy could possibly explain the mistrust and low use of diagnostic tools. A study conducted in Tanzania corroborates these observations [28]. The long held notion of equating fever as a proxy for malaria and the emphasis of malaria as a fatally dangerous disease have also contributed to the tendency for HWs to ignore test results [29]. As a result, a positive test result is often judged as a confirmation of their clinical judgment and a negative test is still regarded as a suspected case of malaria rather than an absence of malaria. Such practices are likely to result in over-treatment with greater cost implications [30]. It is, however assuring that HWs belief in test results are likely to improve with confidence and trust gained through positive outcomes on appropriate prescribing practice. Such change in practice has been observed in a study in Senegal where HW reliance on test results in clinical decision improve with time [31].

The management of <5 children was more likely to conform to the guidelines compared to older patients (Table 4). The high level of appropriate ACT treatment among <5 children is most likely attributable to their higher fever prevalence, and the tendency for HWs to resort to presumptive treatment with ACT perhaps due to practices over the years whereby presence of fever is indicative of malaria. This assertion is supported by the equal proportions of ACT prescribed for under-five children and older patients presenting with fever who had a routine positive test of malaria (Fig. 2).

CHNs and midwives are low in the hierarchy of the HW professional ladder due to their limited scope of practice compared to medical officers. Their relatively huge presence seen in the management of most patients presenting for primary consultations notwithstanding their training on malaria case-management shows major task shifting, an indication of the shortage of medical doctors/medical assistants at the HFs in rural communities. The findings that lower cadre of health staff adhered much more closely to the guidelines compared to medical doctors as found in studies conducted in Kenya and rural Tanzania [8, 32] underscores task shifting as a tool to address shortage of human resource in the health system.

Results from this study also highlights the need for improved laboratory capacity at the primary care level for early and effective management of both malarial and non-malarial fevers. Under-diagnosis of malaria due to low sensitivity of routine diagnostics is a major concern with far-reaching clinical consequences. The “missed opportunity” for early treatment of malaria especially in children could result in severe malaria with high fatality outcome [12]. Likewise, the management of non-malaria febrile illnesses such as sepsis and meningitis require a comprehensive laboratory investigation to narrow down to the cause of fever for effective administration of antibiotic. In the absence of efficient laboratory capacity and proper interpretation of malaria test results based on the guidelines, HWs are likely to continue the wrong prescription of anti-malarial or resort to the use of antibiotics for managing non-malarial febrile illnesses [33, 34].

### Limitations

The interpretation of findings from this study should be interpreted with caution as Hawthorne effect could play an important role considering the context in which data was collected; issues related to observer effects, i.e. a positive change in the behavior of HW resulting in a better than usual performance when observed or monitored closely [35, 36]. This may provide some limitations on the study design. The presence of the study team at the facility before consultation may have influenced HWs performance beyond their usual levels, a further demonstration of their non-compliant behaviour when not observed in their usual practice. The effect may be minimal as observed in other studies which had a member of the study team directly observing HW interactions with patient in the consulting room [37, 38].

## Conclusion

Although a high proportion of <5 children presenting with fever and older patients with uncomplicated malaria received ACT in accordance with the national malaria guidelines, inappropriate treatment with ACT was still high especially among older patients five years after anti-malarial policy change. This was due to poor interpretation of the national malaria treatment guidelines particularly on malaria test result and low quality of routine malaria diagnostics.

Concerted effort towards improving rational use of ACT by HWs is urgently needed if the anticipated benefits of the policy change are to be realized. The Ghana NMCP needs to strengthen the capacity of HWs through regular training [39] and supervision [40] on the malaria guidelines especially on treatment decisions made on the basis of a test result. The capacity of laboratories at the primary health care level needs to be enhanced through regular training for laboratory technicians and development of effective quality control measures.

## Abbreviations

ACT: artemisinin-based combination therapy
AMFm: affordable medicine facility for malaria
IMCI: integrated management of childhood illness
INESS: INDEPTH phase IV effectiveness and safety study
CHPS: community-based health planning services
RDT: rapid diagnostic test
ITN: insecticide-treated net
EDTA: ethylenediaminetetraacetic acid
WHO: World Health Organization
GHS-ERC: Ghana Health Service Ethics Review Committee
KHRC-IEC: Kintampo Health Research Centre Institutional Ethics Committee
LMTS: low malaria transmission season
HMTS: high malaria transmission season
NHIS: National Health Insurance Scheme
CHN: community health nurse
HW: health worker
HF: health facility
NMCP: National Malaria Control Programme
